# JOB CHARACTERISTICS ASSOCIATED WITH INTENT TO QUIT AMONG NURSING HOME EMPLOYEES AND MANAGERS

**DOI:** 10.1093/geroni/igac059.3142

**Published:** 2022-12-20

**Authors:** Katherine Kennedy, David Mohr

**Affiliations:** Providence VA Medical Center, Providence, Rhode Island, United States; VA Boston Healthcare System, Boston, Massachusetts, United States

## Abstract

High turnover and recruitment challenges of nursing home employees and managers is an ongoing concern. This study’s objective was to examine intent to quit among all staff and assess the roles of job characteristics and job satisfaction. Employees and managers within one nursing home chain working in direct patient care or nursing were compared. Data came from the Work, Family, Health Network 18-month follow-up survey in 2012 (Total = 1,000, Managers = 101, Employees = 899). A cumulative logit model controlling for demographics was estimated for lower intent to quit. Herzberg’s Two-Factor Theory of Work Motivation guided the study. Employees scored significantly lower on family-supportive supervisor behaviors (FSSBs), schedule control (SC), and decision authority (DA) than managers. Employees and managers did not differ on job satisfaction, intent to quit, or job demands. Satisfied workers had 5.88 times greater odds of lower intent to quit compared to workers who were neutral or disagreed (p < .0001). DA (OR=0.29) and SC (OR=0.19) were related to higher intent to quit. In contrast, FSSBs (OR = 1.45), safety compliance (OR=1.41), and the combination of high DA with high SC (OR = 1.4) were related to lower intent to quit. Among nursing home staff, lower intent to quit may be achieved through improving job satisfaction, safety culture, the quality of supervision, and job enrichment through more schedule control and decision-making power. It is also imperative to develop strategies to retain those with more education through further research.

